# Distinct RBC alloantibody responses in type 1 interferon-dependent and -independent lupus mouse models

**DOI:** 10.3389/fimmu.2023.1304086

**Published:** 2024-01-15

**Authors:** Kausik Paul, Rosario Hernández-Armengol, June Young Lee, Che-Yu Chang, Tomohiro Shibata, Michifumi Yamashita, Caroline Jefferies, David R. Gibb

**Affiliations:** ^1^ Department of Pathology and Laboratory Medicine, Cedars-Sinai Medical Center, Los Angeles, CA, United States; ^2^ Kao Autoimmunity Institute, Cedars-Sinai Medical Center, Los Angeles, CA, United States; ^3^ Department of Medicine, Division of Rheumatology, Cedars-Sinai Medical Center, Los Angeles, CA, United States; ^4^ Division of Transfusion Medicine, Cedars-Sinai Medical Center, Los Angeles, CA, United States

**Keywords:** RBC alloimmunization, lupus, MRL-*lpr*, type 1 interferons, transfusion, autoimmunity

## Abstract

During transfusion of red blood cells (RBCs), recipients are exposed to both ABO and non-ABO ‘minor’ antigens. RBC donor units and recipient RBCs are not routinely matched for non-ABO antigens. Thus, recipients are exposed to many RBC alloantigens that can lead to RBC alloantibody production and subsequent clinically significant hemolysis. RBC alloantibodies also significantly limit the provision of compatible RBC units for recipients. Prior studies indicate that the frequency of RBC alloimmunization is increased during inflammatory responses and in patients with autoimmune diseases. Still, mechanisms contributing to alloimmune responses in patients with autoimmunity are not well understood. More than half of adult patients with systemic lupus erythematosus (SLE) produce type 1 interferons (IFNα/β) and express IFNα/β stimulated genes (ISGs). Previously, we reported that IFNα/β promote RBC alloimmune responses in the pristane mouse model, which develops a lupus-like phenotype that is dependent on IFNα/β signaling. However, it is unclear whether IFNα/β or the lupus-like phenotype induces alloimmunization in lupus models. Therefore, we tested the hypothesis that IFNα/β promotes RBC alloimmune responses in lupus by examining alloimmune responses in IFNα/β-independent (MRL-*lpr*) and IFNα/β-dependent (pristane) lupus models. Whereas pristane treatment significantly induced interferon-stimulated genes (ISGs), MRL-*lpr* mice produced significantly lower levels that were comparable to levels in untreated WT mice. Transfusion of murine RBCs that express the KEL antigen led to anti-KEL IgG production by pristane-treated WT mice. However, MRL-*lpr* mice produced minimal levels of anti-KEL IgG. Treatment of MRL-lpr mice with recombinant IFNα significantly enhanced alloimmunization. Collectively, results indicate that a lupus-like phenotype in pre-clinical models is not sufficient to induce RBC alloantibody production, and IFNα/β gene signatures may be responsible for RBC alloimmune responses in lupus mouse models. If these findings are extended to alternate pre-clinical models and clinical studies, patients with SLE who express an IFNα/β gene signature may have an increased risk of developing RBC alloantibodies and may benefit from more personalized transfusion protocols.

## 1 Introduction

During allogeneic red blood cell (RBC) transfusion, a recipient is exposed to ABO and non-ABO antigens, such as Kell, Duffy, and Kidd antigens. RBC donors and transfused patients are not routinely matched for antigens other than ABO and Rh(D). Hence, recipients are exposed to as many as 340 non-ABO alloantigens (1). This exposure increases the risk of RBC alloantibody production, which can lead to clinically significant hemolytic transfusion reactions, hemolytic disease of the fetus and newborn during pregnancy, and renal allograft rejection in the transplant setting. RBC alloimmunization also limits the availability of compatible RBC units for anemic patients (2–4). In the 2019 and 2020 Fiscal Years, the FDA reported that hemolytic transfusion reactions due to non-ABO antibodies are one of the leading causes of transfusion-related fatalities in the United States (5, 6). Identifying mechanisms underlying alloantibody production during RBC transfusion would help mitigate the adverse effects of alloimmunization-related hemolysis in RBC recipients.

Prior studies have shown that 3-10% of all transfused recipients develop antibodies against RBC antigens. However, this frequency of alloimmunization is increased in specific patient populations, including chronically transfused patients with hemoglobinopathies (2). Ramsey and Smietana reported that the prevalence of RBC alloantibodies is also elevated in women with autoimmunity (7). Later studies reported elevated frequencies of alloimmunization in patients with specific autoimmune diseases, including systemic lupus erythematosus (SLE) (8–10). Among patients with SLE, approximately 50% have anemia, and as many as 20% of transfused patients produce antibodies against RBC antigens (11, 12). Only patients with sickle cell disease have a higher rate of alloimmunization. However, the underlying molecular and cellular mechanisms that contribute to RBC alloantibody responses in patients with SLE are not well understood.

Multiple studies indicate that inflammation regulates RBC alloimmunization (9, 13–15). Studies in preclinical models indicate that varying inflammatory stimuli have distinct effects on RBC alloantibody formation following transfusion. In murine transfusion models, inflammation caused by influenza and polyomaviruses promotes RBC alloimmunization, while bacteria-derived lipopolysaccharide suppresses alloantibody responses (16–19). Other reports have shown that prolonged storage of murine RBCs can induce inflammatory cytokines, including IL-6, and promote alloimmunization following transfusion (20, 21). Collectively, these studies indicate the significant involvement of specific inflammatory pathways in the regulation of RBC alloimmunization in murine models.

Specifically, pre-clinical studies indicate that type 1 interferons (IFNα/β) regulate alloimmune responses. IFNα/β are inflammatory cytokines first reported as having a key role in anti-viral immunity (22). We previously reported that IFNα/β induced by influenza infection or polyinosinic: polycytidylic acid (poly(I:C)), a viral mimetic, promotes alloimmune responses to transfused RBCs expressing the KEL1 antigen (K1 RBCs) (17, 23). IFNα/β gene signatures are elevated in multiple autoimmune diseases including Sjögren’s syndrome, systemic sclerosis, rheumatoid arthritis, and SLE (24–27). All children with SLE and more than half of adult patients with SLE express IFNα/β gene signatures (28–31), which are associated with increased autoantibody production and disease severity (28, 32–35).

In this study, we evaluated the contribution of IFNα/β inflammation to RBC alloimmune responses in the context of lupus. We previously reported that RBC alloimmune responses are induced in a lupus mouse model, in which injection of pristane oil results in a lupus-like phenotype that is dependent on IFNα/β production (36). However, the extent to which the IFNα/β response or the lupus-like phenotype promotes RBC alloimmunization is not clear. In contrast to the pristane model, MRL-*lpr* mice contain mutations in Fas, a pro-apoptotic gene that facilitates the deletion of auto-reactive lymphocytes. This results in the production of autoantibodies and a lupus-like phenotype that is independent of IFNα/β signaling (37–39). Here, we examined RBC alloimmune responses in these IFNα/β-independent (MRL-*lpr*) and IFNα/β-dependent (pristane) models to test the hypothesis that IFNα/β enhance RBC alloimmune responses in lupus models.

## 2 Materials & methods

### 2.1 Mice

C57BL/6 and MRL-*lpr* mice were obtained from Jackson Laboratories (Bar Harbor, ME, USA). K1 RBC transgenic mice, which express the human KEL glycoprotein (containing the KEL1 antigen) specifically on RBCs, were described previously (23). C57BL/6 and MRL-*lpr* mice were female and 16-20 weeks of age, except for kidney histology experiments as indicated. All pristane-treated mice were injected intraperitoneally with one dose of 0.5 mL pristane (2,6,10,14-tetramethylpentadecane, Sigma-Aldrich, St. Louis, MO, USA) as described previously (36). The Cedars-Sinai Institutional Animal Care and Use Committee approved all mouse protocols.

### 2.2 Transfusion

Blood from K1 and C57BL/6 mice was collected by retro-orbital (RO) bleeding in 12% Citrate Phosphate Dextrose Adenine (CPDA-1, Jorgensen Labs, Melville, NY, USA) and then leuko-reduced using Pall (East Hills, NY, USA) syringe filters. 50μL of leuko-reduced packed RBCs were transfused by tail vein injection to the recipient mice, approximately the murine equivalent of one unit of human RBCs. In some experiments, 100,000 IU of recombinant (rIFNα, Miltenyi Biotec, Bergisch Gladbach, GER) was mixed with K1 RBCs immediately prior to transfusion.

### 2.3 Measurement of inflammatory cytokines

Blood was collected by RO bleeding and serum was obtained following centrifugation. The LEGENDplex Mouse Anti-virus Response Panel was used for cytokine measurement and analysis according to the manufacturer’s instructions (Biolegend, San Diego, CA, USA). Fluorescent beads were acquired with a BD LSRFortessa Cell Analyzer (Becton Dickinson, San Jose, CA, USA).

### 2.4 Renal histology

Kidneys were harvested and preserved in 10% formalin (Medical Chemical Corporation, Torrance, CA, USA). Subsequently, the histopathology lab at Cedars-Sinai prepared slides from paraffin-embedded blocks. Slides were subjected to staining with periodic acid-Schiff (PAS) stain and assessed for scoring by a renal pathologist, (M.Y). The scoring criteria included mesangial expansion and hypercellularity with ratings ranging from none (0) to mild (1), moderate (2), and severe (3).

### 2.5 Anti-KEL alloantibody measurement

Flow cytometric crossmatch was used to measure anti-KEL IgM, IgG and IgG subtypes in mouse serum, as previously described (36). IgM and IgG antibodies were measured 5 and 7-28 days following transfusion, respectively. Secondary antibodies were goat anti-mouse IgM (FITC), IgG (APC), IgG1 (PE), IgG2c (APC), IgG2b (FITC), and IgG3 (BV421) (Jackson ImmunoResearch, West Grove, PA, USA). The anti-KEL IgG graphs represent the peak IgG level, 7-28 days after transfusion.

### 2.6 Post-transfusion recovery

Clearance of transfused RBCs was measured as previously described (36). Briefly, fluorescently-labeled K1 and C57BL/6 RBCs were mixed at a 2:1 ratio and then transfused retro-orbitally into mice previously transfused with K1 RBCs. Naïve K1 transgenic mice, which do not have anti-KEL antibodies, were also transfused to provide a negative control. Mice were phlebotomized 0-4 days after transfusion and fluorescent RBCs were acquired by flow cytometry. The ratio of the percentage of K1 RBCs to the percentage of C57BL/6 RBCs was graphed as post-transfusion recovery.

### 2.7 Analysis of splenocytes and peripheral blood leukocytes by flow cytometry

Spleens were cut using a razor blade and then filtered with a 70 μM nylon mesh. Peripheral blood was collected by RO bleeding. Single-cell suspensions of blood cells and splenocytes were analyzed after RBC lysis with 3-5 mL ACK Lysing Buffer (Quality Biologicals, Gaithersburg, MD). Fc receptor binding of splenocytes was blocked with TruStain FcX (Biolegend, San Diego, CA, USA). Fc receptor blocking was not performed for measuring FcɣRs in peripheral blood leukocytes. Cells were labeled with fluorescently conjugated antibodies, including B220 (RA3-6B2), TCRβ (H57-597), CD11b (M1/70), Ly6C (HK1.4), Ly6G (1A8), FcɣR1 (S18017D), FcɣR2/3 (93), and FcɣR4 (9E9) from Biolegend. Dead cells were excluded by Zombie-NIR or Zombie-Red (Biolegend) staining. Flow cytometry was performed on the Cytek® Northern Lights spectrum flow cytometer (Fremont, CA, USA), and data analysis was conducted using FlowJo v.10.9.0 Software (Tree Star, Ashland, OR, USA).

### 2.8 Quantitative PCR

Monocytes were isolated from splenocytes in single-cell suspension using the EasySep Mouse Monocyte Isolation Kit (StemCell Technologies, Vancouver, BC, Canada). RNA was isolated from monocytes with the Qiagen RNeasy Mini Kit (Hilden, Germany) and reverse-transcribed to cDNA using the Maxima H Minus cDNA Synthesis Master Mix (Thermo Fisher Scientific, Waltham, MA, USA) according to the manufacturer’s instructions. GAPDH, Mx1, ISG15, and IRF7 cDNA were measured by a QuantStudio 5 Real-Time PCR System using PowerUp SYBR Green master mix (Thermo Fisher Scientific). **Supplementary Table 1** contains primer sequences. Target gene expression compared to GAPDH expression was determined using Thermo Fisher Scientific Connect software.

### 2.9 ELISAs

Serum anti-dsDNA IgG was measured using the mouse anti-dsDNA IgG ELISA Kit (Alpha Diagnostic International, San Antonio, TX, USA). For NP-specific antibody responses, mice were immunized with NP-KLH (100 ng/mouse, Biosearch Technologies, Petaluma, CA, USA) emulsified in Imject Alum adjuvant (4mg, 100 μL/mouse, Thermo Fisher Scientific) and boosted after 35 days with NP-KLH (100 ng/mouse). Anti-NP ELISA was performed by coating the ELISA plate with NP-OVA (15ug/ml, Biosearch Technologies) in borate-buffered saline followed by washing and blocking. Serial dilutions of serum samples were added to the coated plates and bound antibodies were detected by HRP-conjugated goat anti-mouse IgG (Jackson ImmunoResearch). TMB substrate (BD OptEIA, Becton Dickinson) was added, and absorbance was measured using a FLUOstar Omega spectrophotometer (BMG LABTECH Inc., NC, USA).

### 2.10 Statistical analysis

Data was analyzed with GraphPad Prism (San Diego, CA, USA). Student’s t-tests and Mann-Whitney *U* tests were used to determine significant statistical differences between two groups of normal and non-normally distributed data, respectively. A one-way ANOVA and Kruskal-Wallis test with a Dunn’s post-test were used to determine the significance between three or more groups of normally and non-normally distributed data, respectively. Anti-KEL antibody quantities and post-transfusion recovery data were analyzed using non-parametric tests. The mean and the standard error of the mean are represented by data bars and error bars, respectively. White circles indicate values from individual mice.

## 3 Results

### 3.1 Autoimmune pathology in MRL*-lpr* and pristane-induced lupus mice

**Figure 1:**
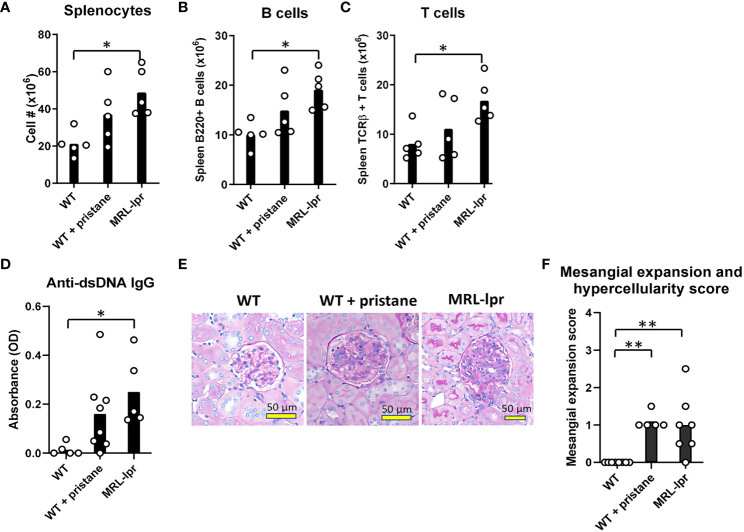
Inflammation and lupus-like phenotypes in pristane-induced and MRL-*lpr* mouse models. Fourteen days before analysis, PrWT (WT + pristane) mice were administered pristane intraperitoneally. **(A-C)** Total number of splenocytes, B cells (B220+), and T cells (TCRβ+) from untreated WT, PrWT, and MRL-*lpr* mice. **(D)** Anti-dsDNA IgG autoantibodies of untreated WT, PrWT, and MRL-*lpr* mice detected in serum by ELISA. **(E)** Periodic acid Schiff stained kidney sections from untreated WT, PrWT, and MRL-*lpr* mice. **(F)** Pathologic scoring of kidney mesangial cell expansion and hypercellularity. **(A-C)** Representative of 2 independent replicated experiments with 5 mice per experimental group; **(D)** Representative of 3 independent replicated experiments with 5-8 mice per experimental group; **(E, F)** Representative of 2 independent replicated experiments with 5-9 mice per experimental group. **(A-C)** PrWT mice administered pristane 14 days or **(D-F)** 6-9 months prior to analysis. Untreated WT and MRL-*lpr* mice are 16-20 weeks **(A-D)** or 6-9 months **(E, F)** of age. *p<0.05, **p<0.01 by One-way ANOVA.

Pristane-induced lupus mice (IFNα/β-dependent) and MRL-*lpr* mice (IFNα/β-independent) were used to determine the impact of lupus-like pathology on RBC alloimmune responses. Administration of pristane, a hydrocarbon oil injected intraperitoneally, leads to toll-like receptor7 (TLR7)-mediated inflammation and lupus-like pathology (40). Pristane treatment of C57BL/6 wildtype (WT) mice caused mortality in 0-20% of mice, as shown in previous studies (data not shown) (41). In contrast to the pristane model, MRL-*lpr* mice contain mutations in Fas, a pro-apoptotic gene expressed in lymphocytes, that cause spontaneous production of autoantibodies and lupus-like pathology that is independent of IFNα/β (37, 38, 42–44). MRL-*lpr* mice were utilized to assess the effect of a lupus-like pathology in an IFNα/β-independent model. Given that the spleen is required for RBC alloimmunization in mice (45), spleen leukocytes were quantified in WT mice, WT mice treated with pristane (PrWT), and MRL-lpr mice. In comparison to untreated WT mice, MRL-*lpr* mice had elevated levels of splenocytes, spleen B and T cells (**Figures 1A–C**). Regarding myeloid cell subsets, MRL-*lpr* mice had a higher number of spleen monocytes, while PrWT mice had higher levels of monocytes and neutrophils compared to WT mice (**Supplementary Figure 1**). In comparison to WT mice, PrWT mice and MRL-*lpr* had increased amounts of lupus-related anti-dsDNA autoantibodies (**Figure 1D**). Kidney histology showed that aged MRL-*lpr* mice (6-9 months of age) and PrWT mice treated with pristane 6-9 months prior to analysis developed mild glomerular mesangial expansion and hypercellularity. Both groups of lupus-like mice exhibited significantly elevated renal pathology scores in comparison to WT mice. However, there were no significant differences between MRL-*lpr* and PrWT mice (**Figures 1E, F**). These data illustrate the presence of lupus-like pathology in both IFNα/β-dependent and -independent lupus models.

### 3.2 Anti-KEL alloimmunization in MRL*-lpr* and PrWT lupus mice

**Figure 2:**
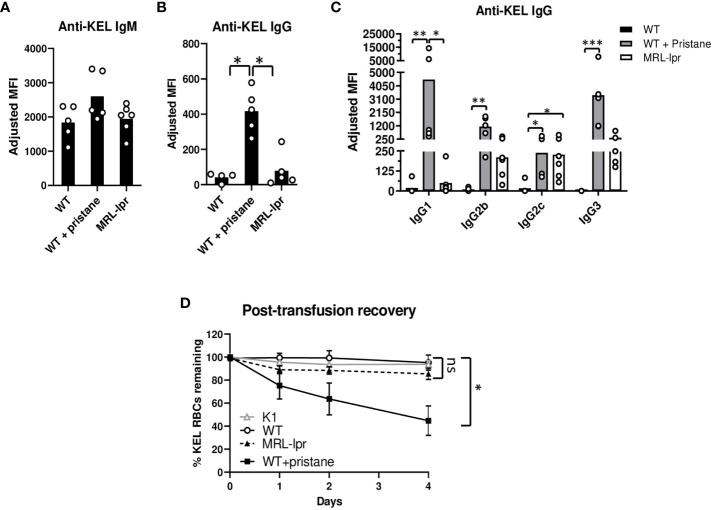
Pristane induces anti-KEL antibodies. K1 RBCs were transfused into WT, PrWT (WT + pristane), and MRL-*lpr* mice, and serum anti-KEL antibodies following K1 RBC transfusion were measured by flow cytometric crossmatch. **(A, B)** Anti-KEL IgM and IgG in WT, PrWT, and MRL-*lpr* mice 5 and 21 days following transfusion, respectively. Adjusted MFI = reactivity of serum with K1 RBCs minus serum reactivity with WT RBCs. **(C)** Anti-KEL IgG subtypes in WT, PrWT, and MRL-*lpr* mice 21 days after transfusion. **(D)** Fluorescently labeled K1 and WT RBCs were mixed at a 2:1 ratio and then transfused retro-orbitally into WT, PrWT, and MRL-*lpr* mice previously transfused with K1 RBCs 35 days earlier. Naïve K1 transgenic mice were also transfused to provide a negative control. Mice were phlebotomized 0-4 days after transfusion and the ratio of K1:WT RBCs in circulation was graphed as post-transfusion recovery. **(A, B)** Representative experiment of more than 3 independent replicated experiments with 4-6 mice per experimental group, **(C)** representative experiment of 2 independent replicated experiments with 5-6 mice per experimental group, and **(D)** representative experiment of more than 3 independent replicated experiments with 5 mice per experimental group, *p<0.05, PrWT (WT + pristane) compared to K1 and WT mice. **(A-D)** *p<0.05, **p<0.01, ***p<0.001 by Kruskal-Wallis test with a Dunn’s post-test. ns, no significant difference.

To investigate RBC alloimmune responses in lupus models, a KEL murine transfusion model, described earlier, was utilized (23). WT mice, PrWT mice injected with pristane 14 days before transfusion, and MRL*-lpr* mice were transfused with leuko-reduced RBCs expressing the KEL1 antigen (K1 mice). The anti-KEL IgM level (5 days following transfusion) and the peak anti-KEL IgG level (21 days after transfusion) were measured by flow cytometric crossmatch. There were no significant differences in levels of anti-KEL IgM. However, PrWT mice produced significantly higher levels of anti-KEL IgG compared to WT and MRL*-lpr* mice (**Figures 2A, B**, **Supplementary Figure 2**). All anti-KEL IgG subtypes including IgG1, IgG2b, IgG2c, and IgG3 were produced in MRL*-lpr* mice and PrWT mice. However, anti-KEL IgG1 was nearly undetectable in MRL*-lpr* mice, compared to high levels in PrWT mice. In comparison to WT mice, PrWT mice had significantly higher levels of each anti-KEL IgG subtype. MRL-*lpr* mice produced elevated amounts of IgG2c compared to WT mice, while levels of other subtypes were comparable between MRL-*lpr* and untreated WT mice (**Figure 2C**).

To examine the impact of anti-KEL antibodies, we measured the degree to which K1 RBCs are removed from peripheral blood circulation. Thirty-five days after the initial transfusion, previously transfused WT, PrWT, and MRL*-lpr* mice were transfused with DiI-labeled K1 RBCs mixed with syngeneic DiO+ C57BL/6 RBCs. To serve as a negative control, K1 mice also received the transfusion. By flow cytometry, the ratio of DiI+ K1 RBCs to DiO+ C57BL/6 syngeneic RBCs in peripheral blood was calculated. Four days following transfusion, approximately half of DiI+ K1 RBCs were removed from peripheral circulation in PrWT mice. In contrast, MRL*-lpr*, WT, and K1 recipients failed to preferentially clear K1 RBCs over WT RBCs (**Figure 2D**).

Given that anti-KEL IgG binding to Fcɣ receptors promotes clearance of K1 RBCs by phagocytosis, we measured the expression of FcɣR1, FcɣR4, and FcɣR2/3 by neutrophils and monocytes in peripheral blood of WT, PrWT, and MRL-*lpr* mice. Monocytes and neutrophils in PrWT mice had elevated expression of FcɣR1 and FcɣR4, compared to WT mice. Neutrophils and monocytes of MRL-*lpr* mice had slightly increased expression of FcɣR2/3, compared to WT and PrWT cells. MRL-*lpr* monocytes also had minimally increased expression of FcgR1 and modestly increased FcɣR4, compared to WT monocytes. The most notable difference in FcɣR expression was the elevated FcgR1 in PrWT monocytes, compared to WT and MRL-*lpr* cells, which may contribute to K1-RBC clearance (**Supplementary Figure 3**). Collectively, these results indicate that different lupus models have distinct alloimmune responses and RBC clearance following RBC transfusion.

### 3.3 MRL*-lpr* lupus mice produce antibodies against a soluble antigen

**Figure 3:**
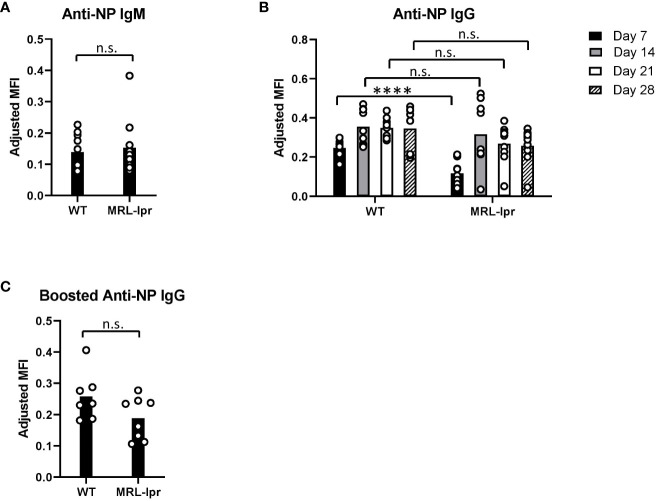
Anti-NP antibodies after immunization with a soluble antigen. **(A, B)** NP-specific IgM and IgG antibodies in NP-KLH (emulsified in alum) immunized WT and MRL-*lpr* mice, measured by ELISA. **(C)** Anti-NP IgG 14 days after an immunization booster, measured by ELISA. Mice were boosted with NP-KLH 35 days after the primary immunization. Representative experiment of 2 independent replicated experiments with 5-10 mice per experimental group. **(B)** ****p<0.0001, n.s., not significant between WT and MRL-*lpr* mice by Mann-Whitney U test.

Since MRL*-lpr* mice produced minimal levels of anti-KEL IgG after RBC transfusion, we examined the degree to which MRL*-lpr* mice respond to immunization with a soluble antigen. After primary immunization with NP-KLH emulsified in alum, we measured anti-NP IgM and anti-NP IgG levels 5 and 7-28 days following immunization, respectively. MRL*-lpr* and WT mice produced comparable levels of anti-NP IgM. MRL-*lpr* and WT mice both produced anti-NP IgG with slightly different kinetics. Anti-NP IgG was elevated in WT mice, compared to MRL-*lpr* mice, 7 days after immunization. However, there were no significant differences in anti-NP IgG between WT and MRL-*lpr* mice 14, 21, and 28 days after immunization.(**Figures 3A, B**). After an immunization booster with NP-KLH 35 days after the initial immunization, there was no significant difference in anti-NP IgG between WT and MRL-*lpr* mice (**Figure 3C**). Although MRL-*lpr* production of anti-NP IgG was delayed, compared to WT mice, these results indicate that MRL-*lpr* mice can generate IgG antibodies against soluble antigens.

### 3.4 Inflammation during the peri-transfusion period in lupus models

**Figure 4:**
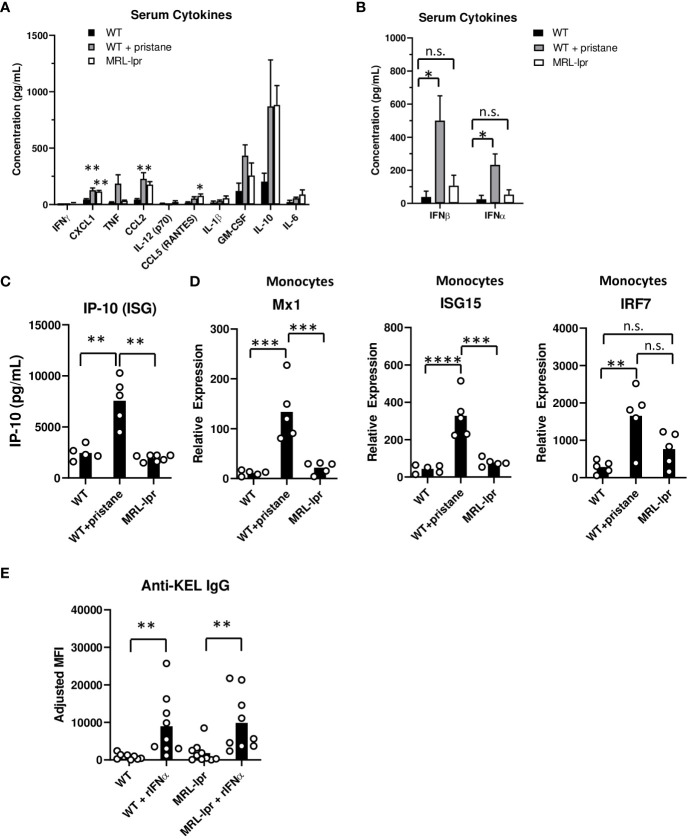
Inflammation and ISG expression in pristane-induced and MRL-*lpr* mouse models. **(A-C)** Serum cytokine levels in WT, PrWT (WT + pristane) and MRL-*lpr* mice were measured by multiplex bead array. **(D)** Mx1, ISG15, and IRF7 expression relative to GAPDH, by spleen monocytes. **(E)** Anti-KEL IgG in serum of WT and MRL-*lpr* mice 7 days after transfusion of K1 RBCs co-transfused with or without recombinant IFNa (rIFNa). Representative of two independent replicated experiments with 9-10 mice per group; **p<0.01 by Kruskal-Wallis test with a Dunn’s post-test. **(A-C)** Representative experiment of 3 independent replicated experiments with 5-7 mice per group. **(A)** * and ** indicate statistical significance compared to untreated WT mice. **(D)** Representative experiment of 2 independent replicated experiments with 5 mice per group. *p<0.05, **p<0.01, ***p<0.001, ****p<0.0001 by One-way ANOVA. ns, no significant difference.

Previous studies showed that inflammation during transfusion of K1 RBCs affects alloimmune responses (17, 23). To assess the inflammatory status, the levels of various serum cytokines were measured at the time of transfusion. The results revealed distinct patterns of cytokine production in the different groups of mice. MRL-*lpr* mice exhibited higher quantities of CCL5 and CXCL1 cytokines compared to untreated WT controls, while PrWT mice produced higher concentrations of CXCL1 and CCL2 compared to WT mice (**Figure 4A**). Notably, PrWT mice displayed elevated levels of IFNβ and IFNα compared to untreated WT mice, while IFNα/β levels in MRL-*lpr* mice did not significantly differ from levels in untreated WT mice (**Figure 4B**). Due to the transient nature of IFNα and IFNβ in murine serum (23), we also measured IFNα/β stimulated genes (ISGs) at the time of transfusion. PrWT mice had high concentrations of the ISG IP-10 in serum, compared to WT and MRL*-lpr* mice (**Figure 4C**). Further examination of the IFNα/β signature was conducted by measuring ISG transcript levels, including Mx1, ISG15, and IRF7, in isolated spleen monocytes by quantitative real-time PCR. Monocytes from PrWT mice expressed increased amounts of Mx1 and ISG15 in comparison to monocytes from WT and MRL-*lpr* mice. Additionally, PrWT monocytes expressed higher levels of IRF7 than monocytes from WT mice (**Figure 4D**). Collectively, these results illustrate the presence of an IFNα/β gene signature in pristane-induced lupus mice and its absence in MRL-*lpr* mice.

Finally, given the low levels of IFNα/β and ISGs in MRL-*lpr* mice, we examined the degree to which IFNα treatment influences RBC alloimmunization in MRL-*lpr* mice. K1 RBCs were co-transfused with or without recombinant IFNα (rIFNα) to WT and MRL-*lpr* mice. rIFNα significantly enhanced anti-KEL IgG production in WT and MRL-*lpr* treated mice, compared to mice transfused without rIFNα (**Figure 4E**). Responses induced by rIFNα peaked one week after transfusion, possibly due to the transient nature of rIFNα treatment. This result indicates that IFNα is sufficient to induce RBC alloimmunization in IFNα/β-independent lupus mice.

## 4 Discussion

Production of IFNα/β and signaling through the IFNα/β receptor contribute to the production of autoantibodies and SLE disease severity. An IFNα/β gene signature is present in greater than half of adult patients and nearly all children with SLE (28–31). In addition, the IFNα/β pathway has been linked to 50% of SLE-related gene variants (46). In accordance with these studies and preclinical findings linking IFNα/β to RBC alloimmunization, we hypothesize that the IFNα/β gene signature contributes to susceptibility to RBC alloimmunization in SLE, possibly independent of disease severity.

Multiple studies have shown that the prevalence of RBC alloimmunization is elevated in patients with autoimmunity, including those with SLE (7–10). Investigation is needed to understand the basic cellular and molecular mechanisms of autoimmune-induced alloimmunization. Several studies indicate that inflammation, including antiviral responses, plays a regulatory role in RBC alloimmune responses (9, 13, 14, 16, 17, 20, 21). We previously reported that K1 RBC transfusion induces RBC alloimmune responses in pristane-induced lupus mice by an IFNα/β-dependent mechanism (36). However, because IFNα/β is also required for development of the pristane-induced phenotype, it was not clear whether IFNα/β or lupus-like pathology enhanced the alloimmune response. To address this in the present study, we tested the hypothesis that IFNα/β induce or enhance RBC alloimmune responses in lupus models by utilizing IFNα/β-independent (MRL-*lpr*) and IFNα/β-dependent (pristane) models. Previous reports showed that IFNα/β signaling is necessary for the development of a lupus-like phenotype in pristane-treated mice (40). Conversely, IFNα/β does not promote autoimmunity in MRL-*lpr* mice. Hron et al. reported that MRL-*lpr* mice that lack the IFNα/β receptor, IFNAR1, surprisingly develop elevated autoantibody levels and more severe end-organ disease, compared to control MRL-*lpr* mice (37). Subsequent studies concluded that IFNα/β either does not affect or protects against lupus pathology in MRL-*lpr* mice (38, 39).

In the current study, compared to PrWT mice, transfused IFNα/β-independent (MRL-*lpr*) mice produced significantly reduced amounts of anti-KEL IgG alloantibodies. Additionally, following re-transfusion with K1 RBCs, IFNα/β-dependent (pristane) mice preferentially cleared transfused K1 RBCs relative to WT RBCS, whereas IFNα/β-independent (MRL-*lpr*) mice did not. This indicates that anti-KEL antibodies formed after the first transfusion can recognize and clear K1 RBCs following subsequent transfusions, possibly through FcɣRs that were significantly elevated in PrWT mice. While memory B cell responses were not directly examined, it is possible that a second transfusion may further increase anti-KEL IgG, leading to preferential clearance of K1 RBCs in PrWT mice. Interestingly, anti-KEL IgM levels did not significantly differ between PrWT, WT, and MRL-*lpr* mice. This suggests that anti-KEL IgM may be regulated by an IFNα/β-independent mechanism. Although MRL-*lpr* mice produced very low amounts of anti-KEL IgG, they were able to produce anti-NP IgM and anti-NP IgG after immunization with a soluble antigen, albeit with slightly delayed kinetics. Finally, IFNα/β-dependent (pristane) mice expressed elevated levels of ISGs compared to IFNα/β-independent (MRL-*lpr*) mice, and infusion of MRL-*lpr* mice with rIFNα induced alloimmunization. Collectively, these results indicate that lupus-like pathology is insufficient to induce alloimmunization. Additionally, given that pristane-induced IFNα/β and rIFNα enhance RBC alloimmunization, IFNα/β may directly promote alloimmunization in lupus mouse models. However, a contributory role of other factors in lupus phenotype development in RBC alloimmunization cannot be ruled out.

Our prior study showed that pristane-treated mice lacking IFNα/β signaling (IFNAR1^-/-^) or production (IRF3/7^-/-^) produced significantly lower levels of anti-KEL IgG after transfusion, compared to PrWT mice (36). We have examined the effect of IFNAR1 blocking antibodies on alloimmunization of PrWT mice that have already developed a lupus-like phenotype. We have found that IFNAR1 blockade a week prior to transfusion and at the time of transfusion does not suppress alloimmunization. This is likely due to the profound and continuous effect of pristane on IFNα/β production and ISG expression prior to antibody treatment.

It was also considered whether pristane may induce anti-KEL IgG production in MRL-*lpr* mice. In one experiment, 80 percent of pristane-treated MRL-*lpr* mice died within 14 days of pristane treatment (data not shown). Pristane is known to cause diffuse alveolar hemorrhage in C57BL/6 mice, as used in this study, resulting in a mortality rate of 10-50% within one month of treatment (41). Whether the elevated mortality of pristane-treated MRL-*lpr* mice is due to diffuse alveolar hemorrhage or other lupus-related sequelae requires further studies. It is also possible that other IFNα/β-inducing stimuli, including poly(I:C), may promote alloimmunization in MRL-*lpr* mice. This possibility should be examined in a future study.

It is worth noting that differences in IFNα/β-induced inflammation are not the only unique aspects between pristane-treated and MRL-*lpr* mice. Lupus-like pathology is acquired in the pristane model, whereas it is genetically induced in MRL-*lpr* mice. Inflammation is initiated in the peritoneum within the first two weeks of pristane treatment, compared to more systemic chronic inflammation in MRL-*lpr* mice (47). Due to the lpr mutation of Fas in B and T cells, MRL-*lpr* mice develop enlarged spleens and lymph nodes containing autoimmune B cells and aberrant T cells (i.e., CD4- CD8- T cells) (48). Given that the spleen is required for RBC alloimmunization in mice (45), altered splenic architecture in MRL-*lpr* mice may alter RBC antigen processing and anti-RBC antibody responses. In addition, while both models produce a comparable array of autoantibodies within 3-4 months of pristane treatment or MRL-*lpr* age, some lupus-like disease manifestations differ. For example, MRL-*lpr* mice develop arthritis and severe nephritis, whereas pristane treatment of C57BL/6 mice does not induce arthritis and leads to a less severe form of nephritis more than 6 months after treatment (39). It is possible that these and other differing manifestations may influence RBC alloimmunization.

This study adds to prior studies indicating that IFNα/β contributes to RBC alloimmunization in pre-clinical models. The initial studies reported IFNα/β-mediated RBC alloimmunity in mice infected with influenza or pre-treated with poly(I:C), a pro-inflammatory viral mimetic (17, 23). It also indicates that the prior report of alloimmune responses in the pristane-induced model resulted from IFNα/β inflammation rather than an IFNα/β-mediated lupus phenotype (36). The current report and the prior one are the first to investigate mechanisms underlying RBC alloimmunization in pre-clinical models of autoimmunity. Future studies should investigate the contribution of IFNα/β to alloimmunization in additional models of lupus and other IFNα/β-contributing autoimmune diseases. They should also address the degree to which IFNα/β mediates RBC alloimmune responses to other RBC antigens.

In summary, we report that a lupus-like phenotype in one pre-clinical model is not sufficient to induce alloimmunization, and IFNα/β gene signatures may be responsible for RBC alloimmunization in lupus mouse models. It is not yet known if these results may extend to other lupus models, which should be examined in future studies. If these results extend to clinical studies, patients with lupus and an IFNα/β signature may have an increased risk of RBC alloimmunization and may be candidates for personalized transfusion protocols such as extended RBC antigen matching prior to transfusion.

## Data availability statement

The original contributions presented in the study are included in the article/**Supplementary Materials**, further inquiries can be directed to the corresponding author.

## Ethics statement

The animal study was approved by Cedars-Sinai Institutional Animal Care and Use Committee. The study was conducted in accordance with the local legislation and institutional requirements.

## Author contributions

KP: Data curation, Formal analysis, Investigation, Writing – original draft. RH-A: Methodology, Writing – review & editing. JL: Formal analysis, Investigation, Methodology, Writing – review & editing. C-YC: Methodology, Writing – review & editing. TS: Investigation, Methodology, Writing – review & editing. MY: Data curation, Formal Analysis, Investigation, Methodology, Writing – review & editing. CJ: Conceptualization, Methodology, Resources, Writing – review & editing. DG: Conceptualization, Funding acquisition, Supervision, Writing – review & editing.
