# Supplementary material for: Distinct RBC alloantibody responses in type 1 interferon-dependent and -independent lupus mouse models
Source: Front Immunol. 2024 Jan 15;14:1304086. doi: 10.3389/fimmu.2023.1304086 (PMC10822987; doi:10.3389/fimmu.2023.1304086)
Supplement: Supplementary file 1 [file DataSheet_1.pdf]

# *Supplementary Material*

## 1 Supplementary Data

**Supplementary Table 1.** PCR primers for quantitative real-time PCR

| mRNA | Forward primer | Reverse Primer |
|---|---|---|
| GAPDH | 5′ CAT CAA GAA GGT GGT GAA GC 3′ | 5′ CCT GTT GCT GTA GCT GTA TT 3′ |
| Mx1 | 5′ GAT CCG ACT TCA CTT CCA GAT GG 3′ | 5′ CAT CTC AGT GGT AGT CAA CCC 3′ |
| ISG15 | 5′ GGT GTC CGT GAC TAA CTC CAT 3′ | 5′ CTG TAC CAC TAG CAT CAC TGT G 3′ |
| IRF7 | 5′ TGC TGT TTG GAG ACT GGC TAT 3′ | 5′ TCC AAG CTC CCG GCT AAG T 3′ |

### 1.1 Supplementary Figures

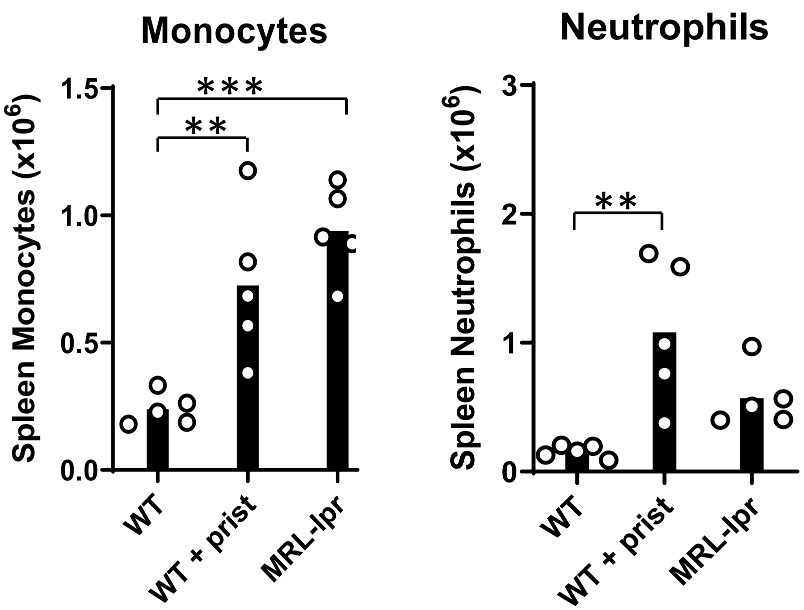


**Supplementary Figure 1. Spleen innate immune cell expansion in pristane-treated and MRL-*lpr* mice.** WT mice were treated with or without pristane 14 days prior to analysis. Quantification of spleen $Ly6C^+$ monocytes and $Ly6G^+$ neutrophils determined by flow cytometry. Representative of 2 independent experiments, 5 mice per group. **p <0.01, ***p < 0.001 by One-way ANOVA.

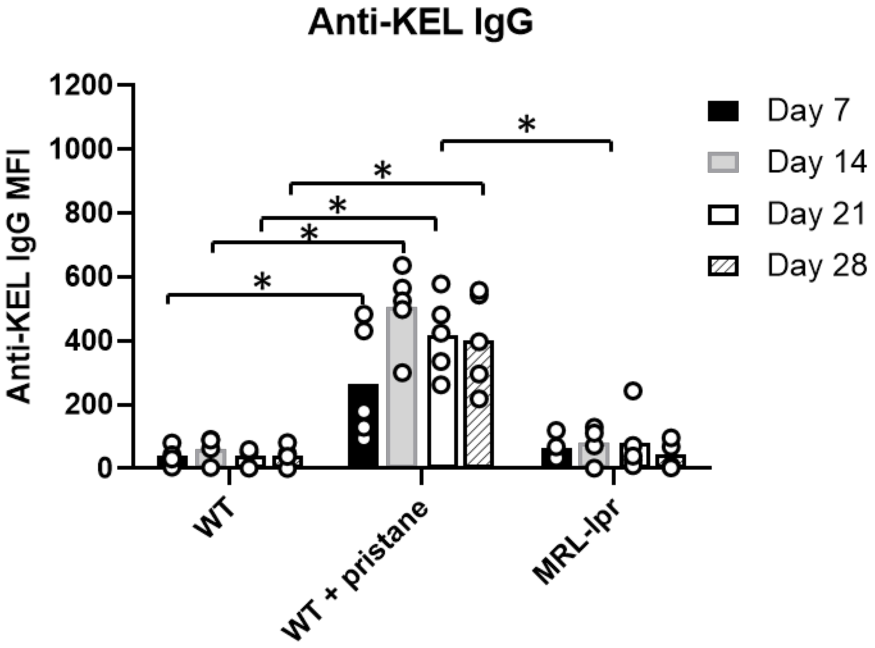


**Supplementary Figure 2. Kinetics of anti-KEL IgG production.** Anti-KEL IgG (7-28 days after transfusion with K1 RBCs) of untreated WT, pristane-treated WT, and MRL-*lpr* mice, measured by flow cytometry crossmatch. 4-5 mice per group. *$p < 0.05$, by Mann-Whitney U test. Representative of 3 independent experiments.

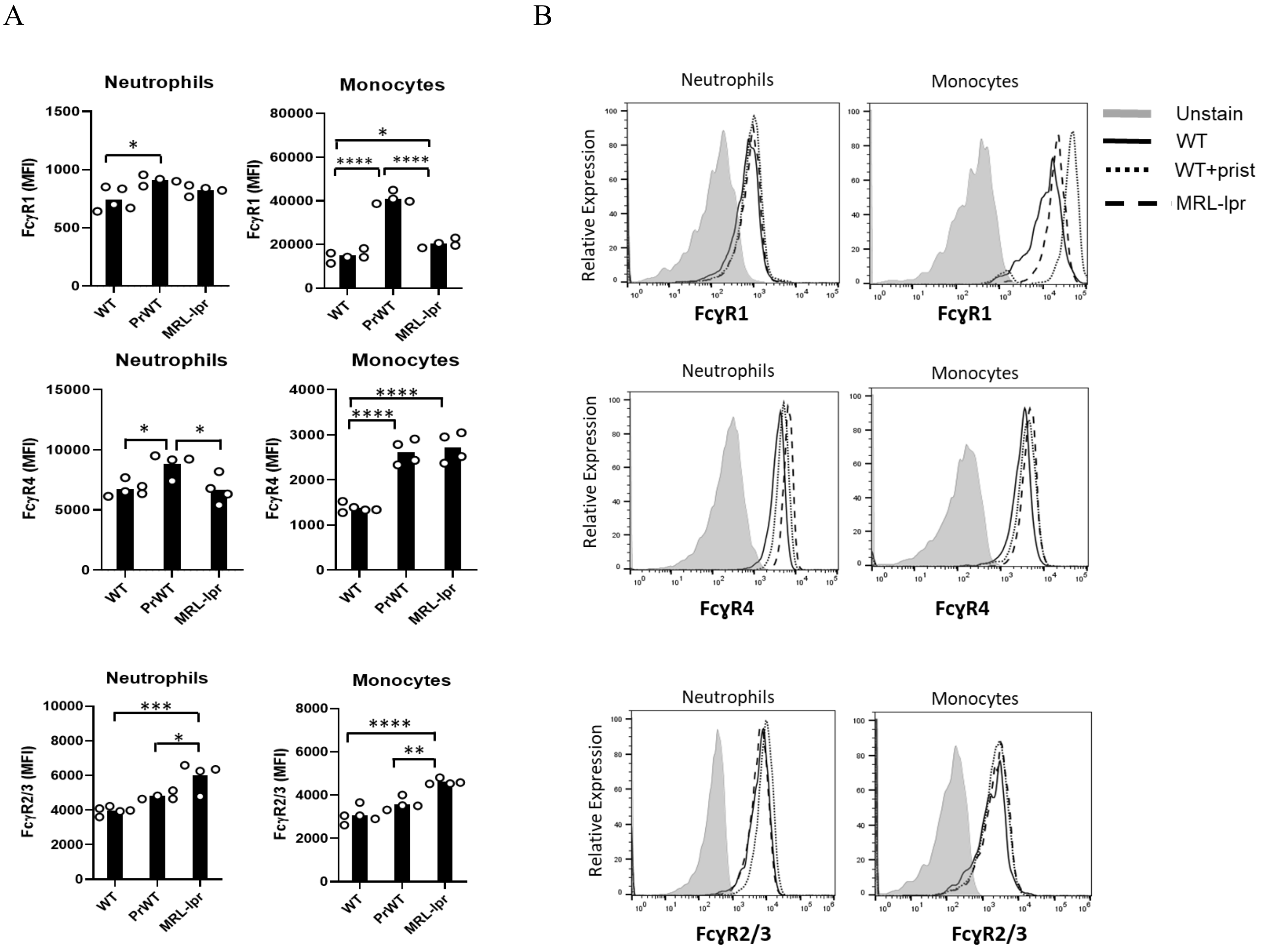


**Supplementary Figure 3. FcɣR expression in lupus mice.** FcɣR1, FcɣR4, and FcɣR2/3 expresion on peripheral blood neutrophils (Ly6G+) and monocytes (CD11b+ Ly6C+) from WT, PrWT, and MRL-lpr mice. A) Summary data and B) Representative histograms measured by flow cytometry. 4-5 mice per group. *$p < 0.05$, **$p < 0.01$, ***$p < 0.001$, ****$p < 0.0001$ by one-way ANOVA with Tukey's post-test.
